# Correction: Polysaccharide-stabilized biogenic CuO/SeO_2_ nanocomposites: green synthesis, physicochemical characterization, and enhanced antioxidant, antimicrobial, and selective anticancer activities

**DOI:** 10.1039/d6ra90095j

**Published:** 2026-08-11

**Authors:** Khaled M. Elattar, Ashraf Elsayed, Alaa Elmetwalli, Mohammed S. El-Hersh, Ayman El-Khateeb, Ahmed A. Zaher, Nesma M. Bayoumy, Mohamed Abdel Salam, Khalid M. Ghoneem, Abdulaziz A. Al-Askar

**Affiliations:** a Unit of Genetic Engineering and Biotechnology, Mansoura University El-Gomhoria St. Mansoura 35516 Egypt khaledelattar2@yahoo.com khaledelattar2@mans.edu.eg; b Botany Department, Faculty of Science, Mansoura University Mansoura 35516 Egypt ashraf-badawy@mans.edu.eg; c Prince Fahad bin Sultan Research Chair for Biomedical Research, University of Tabuk Tabuk Saudi Arabia dr.prof2011@gmail.com aelmetwalli@ut.edu.sa; d Microbial Activity Unit, Department of Microbiology, Soils, Water and Environment Research Institute, Agricultural Research Center Giza 12619 Egypt m.elhersh@yahoo.com; e Department of Agricultural Chemistry, Faculty of Agriculture, Mansoura University Mansoura 35516 Egypt aymanco@mans.edu.eg; f Chemistry Department, Faculty of Science, Mansoura University Mansoura 35516 Egypt smart.zaher@yahoo.com; g Dental Biomaterials Department, Faculty of Oral and Dental Medicine, Delta University for Science and Technology Gamasa Egypt nesma.bayoumy@deltauniv.edu.eg; h Department of Chemistry, College of Science and Technology, Florida A&M University 1530 S. Martin Luther King Blvd, Jones Hall 219, Tallahassee Florida 32307 USA mohamed.salam@famu.edu; i Department of Seed Pathology Research, Plant Pathology Research Institute, Agricultural Research Center (ARC) Giza 12619 Egypt khalidghoneem@arc.sci.eg; j Botany and Microbiology Department, Faculty of Science, King Saud University Riyadh 11451 Saudi Arabia aalaskara@ksu.edu.sa +201010655354

## Abstract

Correction for ‘Polysaccharide-stabilized biogenic CuO/SeO_2_ nanocomposites: green synthesis, physicochemical characterization, and enhanced antioxidant, antimicrobial, and selective anticancer activities' by Khaled M. Elattar *et al.*, *RSC Adv.*, 2026, **16**, 29950–29972, https://doi.org/10.1039/d6ra00556j.

The authors regret that the oxidized forms of kaempferol and eugenol were drawn incorrectly in the graphical abstract and Fig. 1 of the original article.

The corrected versions of the graphical abstract and Fig. 1 are shown below. The oxidized form of eugenol is now represented as 4-allyl-2-methoxycyclohexa-2,5-dien-1-one and the oxidized form of kaempferol is now represented as 2-(4-hydroxybenzoyl)-2,4,6-trihydroxy-3(2H)-benzofuranone.

## Graphical abstract


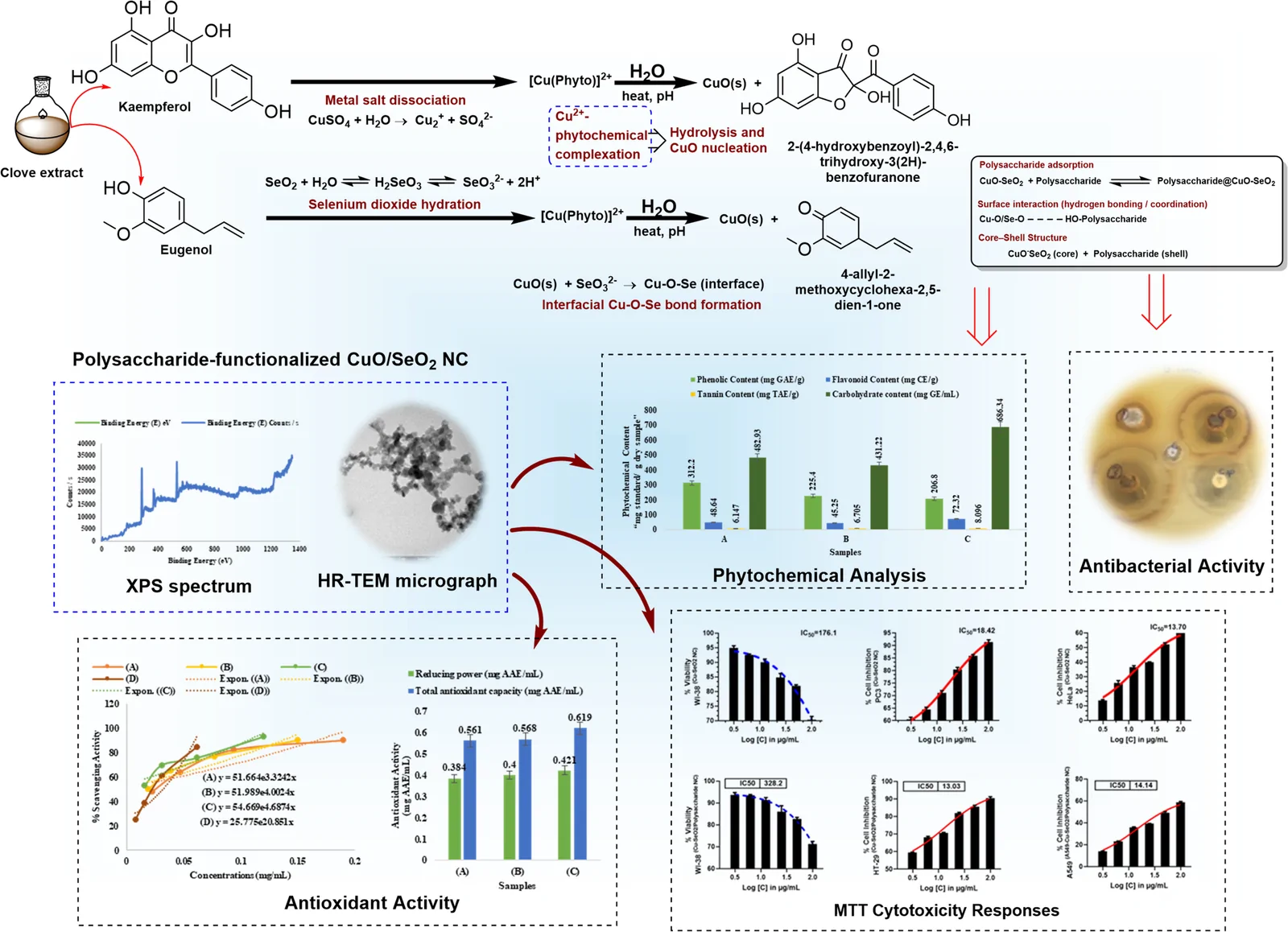
 **Graphical abstract text:** Green-synthesized CuO/SeO_2_ and CuO/SeO_2_/polysaccharide nanocomposites showed stable structures, enhanced antioxidant, antibacterial, and selective anticancer activities, and low toxicity toward normal cells.

## Fig. 1

**Fig. 1:**
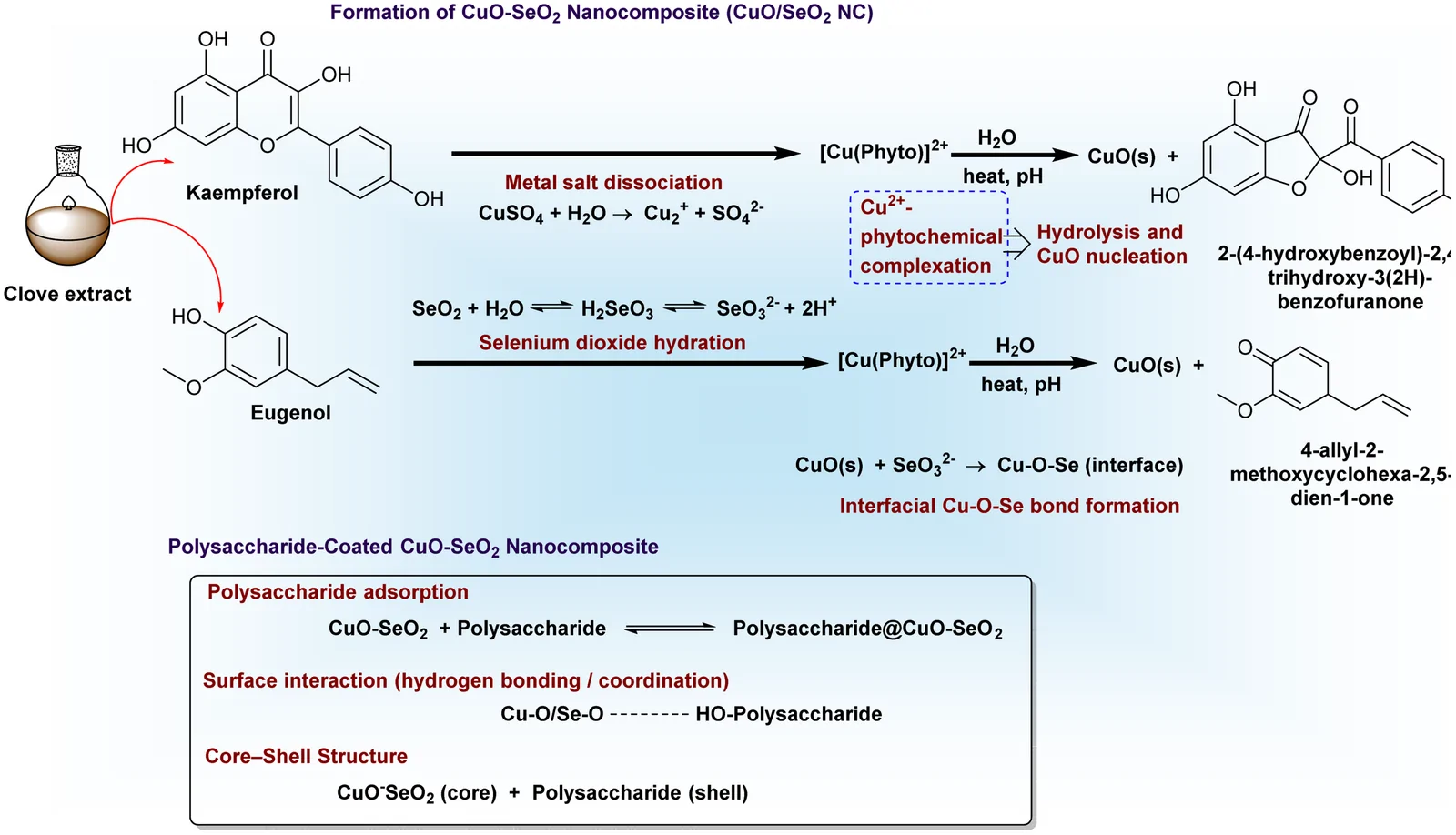
A schematic representation of the proposed green synthesis mechanism for the CuO–/SeO_2_ nanocomposite and its polysaccharide coating architecture is presented. The Cu^2+^ ion complexation, through phytochemicals, and its controlled hydrolysis in the presence of hydrated SeO_2_ species, provide a pathway to CuO nucleation and subsequent interfacial Cu–O–Se bond formation, giving rise to a heterostructured CuO–/SeO_2_ nanocomposite. Further, the adsorption of polysaccharides with ample hydroxyl and carboxyl groups produces a stable core–shell-like CuO/SeO_2_/polysaccharide NC without changing the oxidation states of Cu^2+^ and Se^4+^.

The authors state these changes are limited to the graphical representation of the oxidation products and do not affect the experimental results, interpretation of the data, discussion or overall conclusions of the study.

The Royal Society of Chemistry apologises for these errors and any consequent inconvenience to authors and readers.

